# Sociodemographic Determinants of Reproductive Healthcare Service Use Among Pregnant Women in Pakistan

**DOI:** 10.3390/healthcare13040440

**Published:** 2025-02-19

**Authors:** Zhifei He, Ghose Bishwajit, Fubo Wu

**Affiliations:** 1 School of Politics and Public Administration, Southwest University of Political Science & Law, No. 301, Baosheng Road, Yubei District, Chongqing 401120, China; hezhifei@swupl.edu.cn; 2 Interdisciplinary School of Health Sciences, University of Ottawa, Ottawa, ON K1N 6N5, Canada; brammaputram@gmail.com

**Keywords:** reproductive health, maternal health services, women’s health, sociodemographic determinants, pregnant women

## Abstract

Introduction: Using the essential reproductive care services such as antenatal care (ANC) and skilled birth services are vital for ensuring safe motherhood and controlling maternal and child mortality. There is no recent evidence on the state of using reproductive care services in Pakistan women. The purpose of the cross-sectional study is to explore the timing and frequency of ANC, the hospital and other institutional delivery, the cesarean section (C-section) services and to identify the sociodemographic factors that are associated with the use of these services. Methodology: Using the latest Pakistan Demographic and Health Survey (2017-18 PDHS) for this analysis, the data were collected by face-to-face interviews by trained interviewers, which included 8287 women aged 15–49 years. The data on reproductive services were defined by standard guidelines by the World Health Organization (WHO). Data analyses involved univariate tests and multivariate regression techniques. Results: The percentage of women who attended ANC visits in the first trimester was 62.59%, and those who attended the minimum recommended number of four visits was 49.46%. The percentages of using hospital and C-section services were, respectively, 76.20% and 19.63%. In the regression analysis, place of residence, education, household wealth status, access to using electronic media and learning about family planning from electronic media and before marriage were found to significantly predict the use of ANC and facility delivery services. However, educational and household wealth status stood out as the strongest predictors of all. About half of the women were not having adequate ANC visits and about one-third not making timely ANC contact. More than three-quarters reported choosing to deliver at hospital/other facility, and about one-fifth preferred C-section. Conclusions: The results indicated that, among the predictor of using these services, education and household wealth status were found to have the strongest association, highlighting the role of women’s socioeconomic well-being in availing the basic reproductive healthcare services. Hence, this study suggests that the government and medical institutions should further pay attention to the ANC visits and reduce infant birth mortality rates. Simultaneously, increasing women’s educational opportunities, enhancing women’s socioeconomic well-being and social status, can help improve their health awareness and promote healthy behaviors.

## 1. Introduction

As of 2017, Pakistan is the second-largest country in the South Asian region, with a population of about 197 million. The country has shown promising outcomes in gaining macroeconomic stability and meeting the Millennium Development Goals (MDGs) in recent years [1,2]. Despite that, numerous challenges remain, especially in the healthcare sector. Lack of skilled care providers, research and funding and modern infrastructure are the major barriers [3] that are contributing to challenges for public health, e.g., high maternal and child mortality [4,5]. Maternal and child health indicators are key components of the overall population health metric and human development status of a nation. Yet, developing countries are struggling to meet the goals of reducing maternal and child mortality rates set by the MDGs, to which Pakistan in no exception. According to the most recent estimates from UNICEF, the rate of under-five mortality stands at 74.9 deaths per 1000 live births [6] comparable to that of 78 deaths per 1000 live births in Sub-Saharan Africa [7]. The statistics on maternal mortality rates are equally disheartening. A global analysis published in 2010 on the progress towards MDG 5 reported that Pakistan is one of the top contributors to global maternal mortality worldwide and has a maternal mortality ratio of 297 per 100,000 live births [8]. Similar findings were reported by the Pakistan Demographic and Health Survey (PDHS 2006–2007), with a rate of 276 per 100,000 live births. Pakistan has one of the highest maternal mortality rates in the Asia Pacific region [9]. Given these estimates, the chances of reaching the target stipulated by the Sustainable Development Goals (deaths below 70 for every 100,000 live births by 2030) seem rather uncertain.

Generally speaking, maternal mortality refers to deaths resulting from pregnancy and labor. The standard definition by the International Classification of Diseases (ICD-10) states that it is the maternal mortality death of a woman during pregnancy or within 42 days of termination of pregnancy, irrespective of the duration and site of the pregnancy, from any cause related to or aggravated by the pregnancy or its management [10]. Regardless of the type or cause, pregnancy-related deaths represent a leading contributor to mortality among women of reproductive health worldwide [11], with a vast majority of the deaths taking place in developing countries [10]. Since 1990, efforts to tackle maternal mortality have been reinforced and were highlighted in global agendas, such as the Millennium Development Goals. MDG 5a has been effective since 2000 and was dedicated to the reduction of maternal mortality by 75% between 1990 and 2015 [12]. In the post-MDG era, the Sustainable Development Goals, despite being broader in goals and targets, places special emphasis on women’s health and equality issues [13]. While progress was made in many countries during the MDG period, many still lag behind and experience persistently high maternal and child deaths.

Although these deaths are largely preventable with basic reproductive and maternal healthcare services such as antenatal care and skilled birthing services [14], there exist various supply and demand side barriers. In Pakistan, basic maternal healthcare services are available through public and private clinics and hospitals, along with community-level care centers such as midwifery services [15]. The health centers offer diverse facilities such as family planning, prenatal and assisted birthing. Nonetheless, there are significant barriers in the provision and utilization of these services, including geographic, financial, cultural and motivational factors (low care-seeking behavior) [16,17]. The situation is especially challenging for women owing to the suboptimal rate of participation in the education and labor market that translates to lower socioeconomic empowerment [18]. The sociocultural structure is not conducive enough to promoting gender equality and autonomy among women. The scenario is further exacerbated by the healthcare-related barriers such as lack of qualified care providers, equipment and infrastructure and sparse service centers, especially in remote areas lacking proper transportation facilities [19]. Certain regions in Pakistan are also highly disaster-prone, and seasonal events such as floods make the provision of the basic life-saving services extremely challenging [20]. In a nutshell, the healthcare system in Pakistan, similar to most other South Asian counterparts, is struggling to meet the reproductive care needs for a large segment of the population. Improving this situation will require greater political efforts to resolve the barriers in the delivery and utilization of the services, as well as understanding and addressing the sociodemographic inequalities through generating quality data and studies. For Pakistan, several studies are available on the status of using ANC and skilled delivery services. However, there is no systematic and in-depth research on the use of the broad continuum of reproductive services, starting from the beginning and adequacy of antenatal care to the termination of pregnancy through professional birthing services. Therefore, the rational of this study is to provide a recent and broad picture of the state of maternal healthcare services in Pakistan, emphasizing the role of women’s socioeconomic well-being in accessing basic reproductive health services. In addition, this study suggests that the government and medical institutions should further increase women’s educational opportunities, improve their socioeconomic well-being and social status, enhance their health awareness and promote their healthy behaviors.

## 2. Materials and Methods

### 2.1. Data Source

The study involved a secondary analysis of the Pakistan Demographic and Health Survey (PDHS 2017/18), which is the fourth round of DHS to be conducted in the country. PDHS was implemented by the National Institute of Population Studies (NIPS) in affiliation with the Ministry of National Health Services, Regulations and Coordination, Islamabad, Pakistan. The survey received technical assistance by ICF provided through The DHS Program, a project funded by the United States Agency for International Development (USAID). This is a nationally representative project that surveys adult men, women (15–49 years) and children under five years of age. The survey is cross-sectional in nature and collects data using a standard questionnaire that comprises questions covering demographic, socioeconomic, knowledge of reproductive health and healthcare use-related topics. As these were secondary data, the descriptions on methodology were published already by the DHS team and were not repeated here. The report is available from the website of DHS: National Institute of Population Studies (NIPS) [Pakistan] and ICF. 2019, Pakistan Demographic and Health Survey 2017–18. Islamabad, Pakistan and Rockville, Maryland, USA: NIPS and ICF.

### 2.2. Ethics Approval

The survey was approved by the PDHS review committee. The study was based on the analysis of anonymized secondary data available in the public domain of PDHS; therefore, no additional approval was necessary. All participants gave informed consent prior to taking part in the interview.

### 2.3. Description of Variables

The outcome variables covered two broad categories of reproductive health services: antenatal care and professional birthing services. For antenatal care, we assessed both the timing and frequency of the visits during the last pregnancy. The WHO recommends that the first ANC visit should take place within the first trimester of gestation and at least four visits should be made during the course of the pregnancy [21]. According to these guidelines, these two variables were categorized as (1) the timing of first ANC attendance (within first 3 months = timely, and after 3 months = late) and (2) the total number of ANC attendance (<4 visits = inadequate and 4 or more visits = adequate). The two other outcome variables were place and mode of delivery. These were categorized as place of delivery (hospital/other facility and home delivery) and C-section (yes/no).

The independent variables included the basic demographic and socioeconomic characteristics that were considered important for healthcare seeking behavior. They are as follows: Age groups (15–19, 20–24, 25–29, 30–34, 35–39, 40–44 and 45–49 years); Residence (Urban or Rural); Education (No Education, Primary and Secondary/higher); Wealth quintile (Poorest, Poorer, Middle, Richer and Richest) [22]; Occupation type (None, White collar and Blue collar) [23]; Electronic media, e.g., TV/Radio access (No or Yes); Heard about Family Planning on the TV/Radio (No or Yes); heard about Family Planning before marriage (No or Yes); Husband’s Education (No Education, Primary and Secondary/higher); Spousal Age difference (0–4, 5–9, 10–14 and >15 years) [24].

### 2.4. Statistical Analysis

Data analysis consisted of descriptive statistics, chi-squared bivariate tests and multivariate analyses using Stata version 14 (StataCorp LP, College Station, TX, USA). Firstly, descriptive statistics, along with chi-squared bivariate tests, were performed to describe the distribution sample population for each of the covariates/independent variables. These results were presented as percentages along with frequencies. Chi-squared tests were used to measure the statistical significance of the bivariate association between each of the four outcome variables and the independent variables. Variables that were significant at *p* < 0.05 were selected for multivariate analysis. We performed binary logistic regression to measure the adjusted association between the outcome and independent variables. Four regression models were run for each of the four outcome variables. These results were presented as odds ratios and 95% confidence intervals. Statistical significance was assumed at *p* < 0.05.

## 3. Results

This analysis involved 8287 women aged 15–49 years (Table 1). All women came from urban and rural areas, respectively, and most of them did not receive any education, while a few women receive primary or secondary education. The wealth levels contain the poorest, middle, richer and the richest, and the occupation types included white collar and blue collar. About two-thirds (62.59%) of the women had their first ANC contact within the first trimester, while 49.46% attended the minimum recommended number of four ANC visits. Table 1 also shows that the percentages of using a hospital (or other institutional) and C-section services were, respectively, 76.20% and 19.63%. Women who made timely ANC contact and had adequate visits were more likely to be 25–34 years old, urban residents, had secondary/higher education, from households with higher wealth quintiles, white collar professionals, had access to electronic media, heard about family planning on the TV/radio, heard about family planning before marriage, with husbands having secondary/higher education and had a spousal age difference of 10–14 years. Similar patterns were observed for hospital delivery and C-sectional as well. For instance, the percentages of hospital delivery and C-section were markedly higher among women with secondary/higher education, from the households with higher wealth quintiles and white collar professionals.

The results of multivariate regression analyses on the factors associated with timely and adequate antenatal care utilization are presented in Table 2. Women aged 45–49 years has lower odds of both timely [Odds ratio = 0.512, 95% CI = 0.302, 0.869] and adequate [Odds ratio = 0.551, 95% CI = 0.329, 0.923] antenatal care utilization. Women in rural areas had lowers odds of having adequate ANC visits [Odds ratio = 0.842, 95% CI = 0.752, 0.944]. Having primary and secondary/higher education significantly increased the odds of both. Household wealth quintile also had a significantly positive effect on timely and adequate antenatal care utilization. Employment status was not a significant predictor of the deliveries attended by SBAs. Having access to electronic media [Odds ratio = 1.276, 95% CI = 1.134, 1.434], learning about family planning from electronic media [Odds ratio = 1.252, 95% CI = 1.087, 1.443] and learning about family planning before marriage [Odds ratio = 1.252, 95% CI = 1.087, 1.443] also showed a positive association with adequate use of ANC services but not with early initiation. The husband’s primary and secondary/higher education significantly increased the odds of using both timely and adequate antenatal care utilization. Spousal age difference did not show any significant association with either.

Table 3 shows the results of multivariate regression analyses on the factors associated with hospital and C-section delivery. Age did not show any significant association with hospital delivery; however, those in the age groups of 20–24, 25–29, 30–34 and 35–39 years had higher odds of having a C-section delivery compared with those in the age group of 15–19 years. Rural residents had lower odds of choosing hospital delivery [Odds ratio = 0.838, 95% CI = 0.744, 0.944]. Similar to ANC visits, women’s educational and household wealth status showed the strongest positive association with both hospital and C-section delivery, such that higher educational and wealth status increased the odds of using the respective services. Employment status did not have any significant association with either of the outcomes. Having access to electronic media showed a positive association with both hospital and C-section delivery, whereas learning about family planning from electronic media was associated with C-section delivery [Odds ratio = 1.166, 95% CI = 1.014, 1.341] and learning about family planning before marriage was associated with hospital delivery [Odds ratio = 1.343, 95% CI = 1.139, 1.584]. Husband’s primary and secondary/higher education significantly increased the odds of using hospital delivery but not C-section delivery. Spousal age difference did not show any significant association with the choice of place or mode of delivery.

## 4. Discussion

In the current analysis, we assessed the prevalence of using essential reproductive care services such as the timing and frequency of using antenatal care, hospital/other institutional delivery and C-section. Secondly, we identified the sociodemographic factors that are associated with the use of these services. The findings indicated that about half of the women did not have adequate ANC visits and about one-third not making timely ANC contact. More than three-quarters reported choosing to deliver at hospital/other facility, and about one-fifth preferred a C-section. These findings suggest that there are considerable gaps in the utilization of the basic maternal healthcare services in Pakistan. Similar studies were compared with the current findings for generating a contrasting picture of the current situation. A recent study conducted in Sindh Province found that 57.3% of women had the recommended number of four or more visits, while 53.7% received their first ANC care during the first trimester [25]. In a 2012–13 study, 48.2% of the participants were reported to have delivered at a health facility, compared with 13.3% in the year 1990–91 [25] and 76.20% in the 2017–18 study.

The prevalence of using ANC and birthing services varied significantly across the sociodemographic characteristics of the participants. This was a secondary study, and therefore, the choice of the sample characteristics was limited to the ones available from the dataset. Nonetheless, we did a comprehensive literature review to identify the potential factors that can impact health-seeking behavior among expectant mothers. The variables represent demographic (age); geographic (place of residence); socioeconomic (education, profession and household wealth gradient) and health communication (access to electronic media such as TV and radio and learning about family planning from such media), as well as familial enabling factors (husband’s education and spousal age difference).

Firstly, as the aspect of health awareness enhancement, husbands with a higher education level usually have stronger health awareness and can better understand the importance of the utilization of reproductive health services, thus actively supporting their wives to undergo regular ANC [26]. Secondly, for the aspect of information acquisition ability, educated husbands are more likely to access and understand information about the utilization of reproductive health services, which can help their wives make wiser health decisions and seek professional medical advice when necessary [27]. Thirdly, for the aspect of emotional support, husbands with higher education levels will alleviate their wives’ anxiety and stress and help them better cope with various challenges during pregnancy. Fourthly, for the aspect of healthy behavior demonstration, husbands with higher levels of education usually pay more attention to their own health, which can serve as a good role model, encouraging their wives to develop healthy eating and living habits, which is beneficial for the healthy development of the fetus. In summary, the education level of the husband has a significant positive impact on the utilization of reproductive health services, which can improve the wife’s ANC level and ensure the health of both mother and baby [28,29]. The multivariate analysis produced several interesting insights, including the degree of association between these variables with the outcomes. We found that women’s age was not a strong predictor of using ANC and delivery services, despite the fact that increasing age is often misconstrued as a protective factor against birth complications [30]. Increasing age also serves as a potential indicator of women’s decision-making autonomy, an enabling factor for using maternal healthcare services [31]. Women in rural areas were less likely to access adequate ANC and hospital delivery services, which implies the presence of geographic inequality characteristics of South Asian countries [32,33].

Women’s socioeconomic status was found to be the most significant predictor in the current analysis. Women with higher education and living in higher wealth quintile households were more likely to use ANC and delivery services compared with those with no education and who lived in lower wealth quintile households. Previous studies on healthcare-seeking attitudes and pregnant women and the general population have found similar results [34,35]. The explanation is that higher education is predictive of better health literacy and awareness, which leads to better knowledge and utilization of services. Wealth status is another strong predictor of healthcare-seeking behavior, with higher affordability acting as a facilitator and lower affordability as a barrier to utilizing health services. For countries like Pakistan, where a large segment of the population lives below the poverty line, out-of-expenditure is a serious challenge for the healthcare of poor households, while removing the patient-side expenses is a big challenge for the government as well. Thus, there is a need to find strategic ways to serve the health needs of the most marginalized population in the country. We also observed that women with access to electronic media were generally more likely to use ANC and delivery services. The hypothesis is that exposure to mass media serves as a good source of health communication and thus can greatly enhance women’s knowledge and attitude towards using the services [36]. Women who learnt about family planning before marriage were also more likely to make adequate ANC visits and deliver at a health facility. This finding suggests that improving women’s access to electronic media regarding family planning education could eventually contribute to better use of maternal healthcare services.

In this study, the publicly accessible data for Pakistan were used to assess the current state of reproductive healthcare use in Pakistan. The Pakistan Demographic and Health Survey is a nationally representative survey that collects data on various domains, including demographic, socioeconomic, healthcare-related variables and adult men and women. The reproductive and maternal health domain asks key questions regarding the use of services such as ANC and place of delivery for women of reproductive age (15–49 years). The survey uses a cross-sectional design that limits the assessment of certain statistical parameters such as causation. Nonetheless, the cross-sectional analysis provides a good understanding of the status quo scenario, such as the prevalence of using reproductive services and temporal associations between the use of these services and the sociodemographic characteristics. Research data on the reproductive health indicators can aid in the advancement of the understanding of the social determinants of health, as well as evidence-led policymaking, targeting better population health outcomes.

There are several strengths and limitations to report. This study used a large nationally representative dataset. The data were collected in 2017–18 and thus reflect a very recent picture of using reproductive healthcare services. There is also a scarcity of studies that have reported both ANC and delivery service utilization in the same analysis. In this study, we reported both in order to provide a clearer picture of the situation for further research and policymaking purposes.

### Limitations of the Present Study

As mentioned earlier, this was a secondary study, which limited the choice of the sample characteristics. For this reason, some critical variables such as cultural and infrastructure-related factors were not included in the analysis. Data on service utilization were self-reported and therefore can be subject to recall bias. Lastly, the survey was cross-sectional, and hence, the associations are not guaranteed to establish any causal relationship.

## 5. Conclusions

This study reveals that about half of the pregnant women in Pakistan do not make adequate ANC visits and about one-third do not make timely ANC contact, while more than three-quarters report choosing to deliver at hospital/other facility and about one-fifth prefer C-sections. As such, there are considerable gaps in the utilization of basic maternal healthcare services. Moreover, there were significant inequalities in the use of these services among different population subgroups. Most prominent among them were education and wealth quintiles, such that women with higher illiteracy and wealth status were significantly more likely to avail upon these services compared to those in the lower education and wealth category. Based on these findings, it is recommended that the healthcare system makes special effort to minimize the socioeconomic disparities in the use of vital maternal healthcare services. This is important both from the perspective of meeting the global health agenda (such as SDGs), as well as strengthening the national development efforts by promoting population health. In addition, it is recommended to strengthen the overall level of systematic maternal healthcare services management in Pakistan. Firstly, expanding the educational opportunities for fertile women in Pakistan and improving their education level, especially the health education level. Secondly, enhancing the prenatal examination knowledge of pregnant women and increasing their ANC frequency in Pakistan. Thirdly, increasing the husband’s education level and enhancing his awareness of the utilization of basic maternal healthcare services. Fourthly, the Pakistani government, especially the Pakistani health department, should provide more effective policy support for fertile women.

Future research will focus on the health management and promotion of key populations, including basic maternal health services, in underdeveloped countries or regions.

## Figures and Tables

**Table 1:** Percentage of the use of four types of maternal health services (*n* = 8287).

	Timing of First ANC Contact		Number of ANC Contacts		Place of Delivery		C-Section	
	Late 37.41%	Timely 62.59%	<Four 50.54%	4/More 49.46%	Home 32.80%	Hospital 76.20%	No 80.37%	Yes 19.63%
Age								
15–19	103	161	184	133	111	206	287	30
%	39.02	60.98	58.04	41.96	35.02	64.98	90.54	9.49
20–24	503	847	818	743	493	1069	1287	275
%	37.26	62.74	52.4	47.6	31.56	68.44	82.45	17.61
25–29	749	1356	1166	1268	776	1658	1911	523
%	35.58	64.42	47.9	52.1	31.88	68.12	78.55	21.49
30–34	645	1123	923	1123	608	1438	1572	474
%	36.48	63.52	45.11	54.89	29.72	70.28	76.87	23.17
35–39	438	679	718	628	474	872	1099	247
%	39.21	60.79	53.34	46.66	35.22	64.78	81.77	18.35
40–44	140	198	263	172	176	259	367	68
%	41.42	58.58	60.46	39.54	40.46	59.54	84.56	15.63
45–49	51	35	116	31	80	67	133	14
%	59.3	40.7	78.91	21.09	54.42	45.58	90.48	9.52
*p*-value	0.017		<0.001		<0.001		<0.001	
Residence								
Urban	1022	2422	1368	2370	812	2926	2762	976
%	29.67	70.33	36.6	63.4	21.72	78.28	74.01	26.11
Rural	1607	1977	2820	1728	1906	2643	3893	656
%	44.84	55.16	62.01	37.99	41.9	58.1	85.6	14.42
*p*-value	<0.001		<0.001		0.004		<0.001	
Education								
No Education	1569	1508	2922	1255	1997	2181	3795	383
%	50.99	49.01	69.95	30.05	47.8	52.2	90.9	9.17
Primary	396	619	513	588	317	784	900	201
%	39.01	60.99	46.59	53.41	28.79	71.21	81.74	18.26
Secondary/higher	664	2272	753	2255	404	2604	1960	1048
%	22.62	77.38	25.03	74.97	13.43	86.57	65.25	34.84
*p*-value	<0.001		<0.001		<0.001		<0.001	
Wealth quintile								
Poorest	715	479	1443	384	1035	792	1715	112
%	59.88	40.12	78.98	21.02	56.65	43.35	93.87	6.13
Poorer	698	762	1237	626	828	1035	1679	184
%	47.81	52.19	66.4	33.6	44.44	55.56	90.17	9.88
Middle	545	926	799	823	461	1161	1319	303
%	37.05	62.95	49.26	50.74	28.42	71.58	81.37	18.68
Richer	431	998	499	987	269	1217	1053	433
%	30.16	69.84	33.58	66.42	18.1	81.9	71.0	29.14
Richest	240	1234	210	1278	125	1364	889	600
%	16.28	83.72	14.11	85.89	8.39	91.61	59.78	40.30
*p*-value	<0.001		<0.001		<0.001		<0.001	
Occupation type								
None	2247	3832	3613	3569	2340	4843	5787	1396
%	36.96	63.04	50.31	49.69	32.58	67.42	80.63	19.43
White collar	93	268	109	260	66	303	241	128
%	25.76	74.24	29.54	70.46	17.89	82.11	65.49	34.69
Blue collar	289	299	466	269	312	423	627	108
%	49.15	50.85	63.4	36.6	42.45	57.55	85.31	14.69
*p*-value	<0.001		<0.001		<0.001		<0.001	
Electronic media access								
No	1215	1292	2292	1082	1570	1804	3044	330
%	48.46	51.54	67.93	32.07	46.53	53.47	90.25	9.78
Yes	1414	3107	1896	3016	1148	3765	3611	1302
%	31.28	68.72	38.6	61.4	23.37	76.63	73.59	26.50
*p*-value	<0.001		<0.001		<0.001		<0.001	
Heard about FP in TV/Radio								
No	2231	3278	3709	2967	2412	4265	5567	1110
%	40.5	59.5	55.56	44.44	36.12	63.88	83.41	16.62
Yes	398	1121	479	1131	306	1304	1088	522
%	26.2	73.8	29.75	70.25	19.01	80.99	67.75	32.42
*p*-value	<0.001		<0.001		<0.001		<0.001	
Heard about FP before marriage								
No	2316	3460	3745	3213	2471	4488	5708	1251
%	40.1	59.9	53.82	46.18	35.51	64.49	82.09	17.98
Yes	311	938	439	884	245	1078	942	381
%	24.9	75.1	33.18	66.82	18.52	81.48	71.26	28.80
*p*-value	<0.001		<0.001		<0.001		<0.001	
Husband’s Education								
No Education	793	758	1595	609	1100	1104	1992	212
%	51.13	48.87	72.37	27.63	49.91	50.09	90.38	9.62
Primary	410	541	640	479	395	724	972	147
%	43.11	56.89	57.19	42.81	35.3	64.7	86.86	13.14
Secondary/higher	1385	3048	1892	2961	1185	3669	3612	1242
%	31.24	68.76	38.99	61.01	24.41	75.59	74.52	25.59
*p*-value	<0.001		<0.001		<0.001		<0.001	
Age difference								
0–4	1326	2226	2116	2073	1399	2791	3374	816
%	37.33	62.67	50.51	49.49	33.39	66.61	80.56	19.47
5–9	836	1403	1355	1315	857	1813	2130	540
%	37.34	62.66	50.75	49.25	32.1	67.9	79.92	20.22
10–14	298	553	457	507	298	666	775	189
%	35.02	64.98	47.41	52.59	30.91	69.09	80.39	19.61
≥15	128	167	198	155	125	228	296	57
%	43.39	56.61	56.09	43.91	35.41	64.59	83.85	16.15
*p*-value	<0.001		<0.001		<0.001		<0.001	

Descriptive statistical analyses were adopted in Table 1. (*p* < 0.001, ANC = antenatal care).

**Table 2:** Factors associated with antenatal care (early and adequate visits) in Pakistan in 2017–2018.

Variables	Early Visit	Adequate Visit
Age groups (15–19)	1	1
20–24	0.926 [0.696, 1.232]	1.012 [0.769, 1.333]
25–29	0.901 [0.683, 1.189]	1.095 [0.838, 1.431]
30–34	0.841 [0.635, 1.114]	1.212 [0.924, 1.589]
35–39	0.844 [0.631, 1.129]	0.978 [0.738, 1.294]
40–44	0.872 [0.615, 1.238]	0.843 [0.601, 1.183]
45–49	0.512 * [0.302, 0.869]	0.551 * [0.329, 0.923]
Residency (Urban)	1	1
Rural	0.924 [0.821, 1.040]	0.842 ** [0.752, 0.944]
Education (None)	1	1
Primary	1.235 ** [1.056, 1.444]	1.608 *** [1.383, 1.870]
Secondary/higher	1.862 *** [1.613, 2.150]	2.363 *** [2.062, 2.708]
Wealth quintile (Poorest)	1	1
Poorer	1.379 *** [1.170, 1.625]	1.360 *** [1.160, 1.594]
Middle	1.841 *** [1.541, 2.200]	2.019 *** [1.701, 2.397]
Richer	2.138 *** [1.750, 2.612]	3.030 *** [2.499, 3.675]
Richest	3.902 *** [3.094, 4.921]	7.036 *** [5.588, 8.859]
Employment (None)	1	1
White collar	1.088 [0.835, 1.419]	1.192 [0.910, 1.560]
Blue collar	0.886 [0.737, 1.065]	1.009 [0.841, 1.210]
Access to electronic media (No)	1	1
Yes	1.062 [0.939, 1.200]	1.276 *** [1.134, 1.434]
Heard of FP in electronic media (No)	1	1
Yes	1.104 [0.956, 1.275]	1.252 ** [1.087, 1.443]
Heard of FP before marriage (No)	1	1
Yes	1.320 *** [1.135, 1.534]	1.165 * [1.006, 1.348]
Husbands education (None)	1	1
Primary	1.038 [0.873, 1.232]	1.252 ** [1.059, 1.479]
Secondary/higher	1.078 [0.938, 1.239]	1.424 *** [1.247, 1.626]
Age difference (0–4 years)	1	1
5–9	0.949 [0.845, 1.066]	0.922 [0.824, 1.033]
10–14	1.060 [0.898, 1.253]	1.022 [0.868, 1.203]
≥15	0.981 [0.761, 1.264]	1.160 [0.906, 1.487]

Number are odds ratios with 95% confidence intervals in brackets. * *p* < 0.05, ** *p* < 0.01 and *** *p* < 0.001.

**Table 3:** Factors associated with skilled birth service utilization (hospital delivery and C-section) in Pakistan in 2017–2018.

Variable	Hospital/Other Institutional Delivery	C-Section
Age groups (15–19)	1	1
20–24	0.971 [0.738, 1.277]	1.706 * [1.126, 2.585]
25–29	0.867 [0.665, 1.131]	1.920 ** [1.279, 2.881]
30–34	0.950 [0.725, 1.244]	2.061 *** [1.370, 3.099]
35–39	0.854 [0.647, 1.127]	1.769 ** [1.162, 2.693]
40–44	0.782 [0.564, 1.084]	1.607 [0.989, 2.609]
45–49	0.655 [0.426, 1.006]	1.414 [0.680, 2.943]
Residency (Urban)	1	1
Rural	0.838 ** [0.744, 0.944]	1.070 [0.936, 1.224]
Education (None)	1	1
Primary	1.453 *** [1.241, 1.700]	1.432 *** [1.169, 1.754]
Secondary/higher	2.364 *** [2.029, 2.754]	2.287 *** [1.930, 2.712]
Wealth quintile (Poorest)	1	1
Poorer	1.258 ** [1.093, 1.447]	1.268 [0.980, 1.642]
Middle	1.954 *** [1.658, 2.304]	2.052 *** [1.585, 2.658]
Richer	2.762 *** [2.265, 3.370]	3.056 *** [2.332, 4.005]
Richest	5.113 *** [3.966, 6.592]	4.115 *** [3.095, 5.470]
Employment (None)	1	1
White collar	1.133 [0.836, 1.536]	1.236 [0.964, 1.585]
Blue collar	1.059 [0.893, 1.257]	1.191 [0.942, 1.505]
Access to electronic media (No)	1	1
Yes	1.246 *** [1.108, 1.402]	1.409 *** [1.205, 1.646]
Heard of FP in electronic media (No)	1	1
Yes	1.019 [0.870, 1.193]	1.166 * [1.014, 1.341]
Heard of FP before marriage (No)	1	1
Yes	1.343 *** [1.139, 1.584]	0.998 [0.860, 1.159]
Husbands education (None)	1	1
Primary	1.278 ** [1.089, 1.499]	0.872 [0.686, 1.108]
Secondary/higher	1.216 ** [1.071, 1.381]	1.115 [0.926, 1.344]
Age difference (0–4 years)	1	1
5–9	1.016 [0.906 1.139]	1.008 [0.884, 1.149]
10–14	1.019 [0.863, 1.204]	0.923 [0.763, 1.115]
≥15	1.234 [0.965, 1.579]	1.181 [0.862, 1.617]

Numbers are odds ratios with 95% confidence intervals in brackets. * *p* < 0.05, ** *p* < 0.01 and *** *p* < 0.001.

## Data Availability

The datasets supporting the conclusions are available from the first author (hezhifei@swupl.edu.com) and the second author (brammaputram@gmail.com) upon reasonable request.
